# Scanning Acousto‐Optoelectric Spectroscopy on a Transition Metal Dichalcogenide Monolayer

**DOI:** 10.1002/adma.202402799

**Published:** 2024-10-24

**Authors:** Emeline D. S. Nysten, Matthias Weiß, Benjamin Mayer, Tobias M. Petzak, Ursula Wurstbauer, Hubert J. Krenner

**Affiliations:** ^1^ Physikalisches Institut Universität Münster Wilhelm‐Klemm‐Straße 10 48149 Münster Germany; ^2^ Lehrstuhl für Experimentalphysik I Universität Augsburg Universitätsstraße 1 86159 Augsburg Germany

**Keywords:** carrier localization, charge carrier dynamics, defects, surface acoustic wave, transition metal dichalcogenides, tungsten diselenide

## Abstract

The charge carrier dynamics are investigated by surface acoustic waves (SAWs) inside a WSe_2_ monolayer on LiNbO_3_ by scanning acousto‐optoelectric spectroscopy. A strong enhancement of the PL emission intensity is observed almost over the entire area of the flake. This enhancement increases with increasing amplitude of the wave and is especially strong at or in the vicinity to defects. The latter is attributed to the SAW‐driven Poole–Frenkel activation of trapped charge carriers bound to trapping sites at these defects. In addition, the PL intensity exhibit clear periodic modulations at the SAW's frequency *f*_SAW_ and at 2 *f*_SAW_. These modulations are clear and unambiguous fingerprints of spatio‐temporal carrier dynamics driven by the SAW. These occur on sub‐nanosecond timescales which are found in good agreement with calculated exciton dissociation times. Mapping and analyzing both effects, this study shows that scanning acousto‐electric spectroscopy provides a highly sensitive and local contact‐free probe which uncovers distinct local features not resolved by conventional quasi‐static photoluminescence techniques. The method is ideally suited to study carrier transport in 2D and other types of nanoscale materials and to reveal dynamic exciton modulation, and carrier localization and activation dynamics in the technologically important megahertz to gigahertz frequency range.

## Graphical abstract

Combining time‐resolved optical spectroscopy and surface acoustic waves provide a powerful tool for investigating charge carrier dynamics in 2D materials. When interacting with the 2D semiconductor WSe_2_, dynamic acoustic Poole–Frenkel activation of traps enhances the optical emission efficiency. The sub‐nanosecond modulation of the optical signal serves as a sensitive local probe of defects and charge carrier trapping sites.

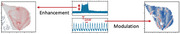

## 1 Introduction

Since the discovery of graphene ≈20 years ago, the field of 2D materials has become one of the most active at the forefront of modern solid‐state physics. Since then, many other 2D crystals and van der Waals structures are studied in detail revealing often properties differing dramatically from those of their bulk counterparts.^[^ ^1^, ^2^, ^3^ ^]^ In this vibrant field, semiconducting transition metal dichalcogenides (TMDCs) have been widely investigated because of their moderate electronic bandgap and outstanding optical properties, which are dominated by strongly interacting interband charge excitations called excitons.^[^ ^3^, ^4^, ^5^ ^]^ These Coulomb‐bound electron–hole pairs have large binding energy due to the strong quantum confinement in 2D and the reduced dielectric screening.^[^ ^4^, ^5^ ^]^ Thanks to these attributes, 2D TMDCs enable the manipulation of the excitons at room temperature using an external electric field. Surface acoustic waves (SAWs) are a versatile tool opening cross‐disciplinary applications in nanoscience.^[^ ^6^ ^]^ When excited on piezoelectric substrates, SAWs provide a contact free method to drive acousto‐electric currents in low dimensional semiconductor systems^[^ ^7^, ^8^, ^9^, ^10^ ^]^ including 2D materials.^[^ ^11^, ^12^, ^13^, ^14^ ^]^ Moreover, SAWs manipulate, control and probe spatio‐temporal dynamics of photogenerated carriers inside semiconductor nanostructures such as quantum wells^[^ ^15^, ^16^, ^17^ ^]^, nanowires^[^ ^18^, ^19^, ^20^, ^21^ ^]^ and quantum dots.^[^ ^22^, ^23^, ^24^ ^]^ SAW spectroscopy has also been used to study the impact of dynamic strain on the photoluminescence (PL) emission in piezoelectric CVD‐grown odd‐layer MoS_2_ on Sapphire‐on‐LiNbO_3_.^[^ ^25^ ^]^ More recently, SAWs have shown to improve the properties of SnS_2_‐ and MoS_2_‐based photodetectors^[^ ^26^, ^27^ ^]^ and they have been used to manipulate the emission of trions and excitons inside exfoliated WSe_2_ and MoSe_2_ monolayers.^[^ ^28^, ^29^, ^30^ ^]^ In these studies, the quenching of the PL emission due to exciton dissociation under the SAW electric field was observed^[^ ^28^, ^29^ ^]^ However, no direct measurement of the SAW‐induced dynamics in the time‐domain is reported in these studies and only obtained indirectly. In addition, time‐ and position dependent experiments on monolayer^[^ ^30^ ^]^ and bilayer TMDCs^[^ ^31^ ^]^ revealed exciton transport, which is not driven by the SAW's electric field but mediated by its propagating strain.

In this work, we employ acousto‐optoelectric spectroscopy (AOES)^[^ ^18^ ^]^ on monolayer WSe_2_ transferred onto a LiNbO_3_ SAW device at room temperature. We find a clear periodic modulation of the optical emission of the WSe_2_ in the time domain. The observed period is directly determined by the frequency of the SAW. These oscillations set in when the exciton dissociation driven by the SAW's electric field falls below the PL decay time. Calculation using realistic parameters confirm that this onset occurs at accessible electric fields. Thus, our observation marks an unambiguous proof of the presence of SAW‐driven carrier dynamics in the 2D semiconductor. In addition, we find a pronounced enhancement of the time‐averaged PL intensity under SAW modulation. This initially unexpected observation can be explained and modeled by a Poole–Frenkel activation by the dynamic electric field of the SAW. We employ scanning AOES on the full flake and find that both effects, the dynamic PL emission and the enhancement of the time‐averaged emission are highly position dependent and provide direct insight into ubiquitous carrier localization in the 2D semiconductor. We show that this mapping AOES can be readily employed to resolve trapping sites which are not observed in conventional scanning PL measurements. Moreover, time‐ and position‐dependent AOES reveal directional carrier activation and transport, and the inhibition of transport by local defects.

## 2 Results

**Figure 1:**
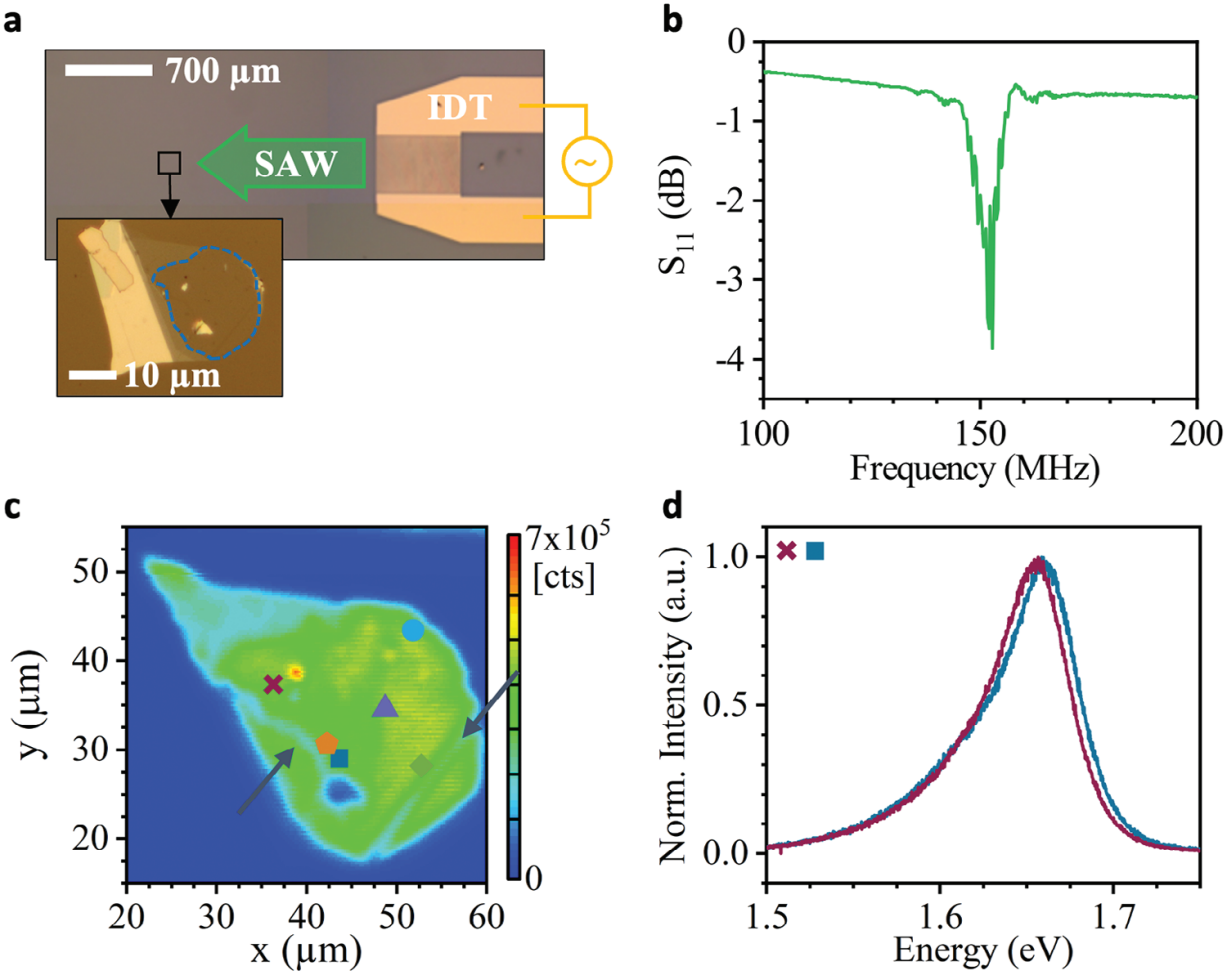
Sample layout and precharacterization – a) Optical microscope image of the fabricated SAW‐hybrid device, Sample 1. A monolayer of WSe_2_ is transferred onto a LiNbO_3_ YZ‐cut substrate on the left of an IDT exciting the SAW. b) Scattering parameter S_11_ quantifying the electrical reflection of the IDT. SAWs are excited at frequencies ≈152 MHz. c) False color map of the integrated PL intensity of the WSe_2_ monolayer. The colored symbols show the position at which subsequent analyses were taken. d) Typical static PL spectra of the exciton emission in WSe_2_ at the positions indicated in c by the dark blue square and violet cross.

We start by introducing the SAW‐hybrid device layout and its main components. Throughout this article, we present results of two samples, which we refer to as Sample 1 and Sample 2 in the following. **Figure** **1**a shows an optical microscope image of Sample 1. Complementary information on Sample 2 can be found in the Experimental Section and Supporting Information. For both samples, a WSe_2_ monolayer flake was isolated using an established exfoliation method and transferred onto a LiNbO_3_ YZ‐cut substrate using a PDMS stamp. The flake was not encapsulated in hBN to study the native interactions with the LiNbO_3_ SAW. This ensures that the coupling is dominated by the electric field component of the SAW and strain coupling is negligible.^[^ ^24^, ^28^, ^30^, ^32^ ^]^ The size of the monolayer is outlined by the blue dashed line and is roughly 20 × 20 µm^2^. SAWs are excited by applying a radio frequency (rf) signal to an interdigital transducer (IDT) placed at the right of the WSe_2_ flake. The electrical characterization of the IDT is conducted using a vector network analyzer (VNA) and the magnitude of the reflective scattering parameter S_11_ is plotted in Figure 1b. From these data, we extract a central frequency of the SAW *f*_SAW_ = 152 MHz and a bandwidth of 10 MHz, matching the lithographically defined wavelength λ_SAW_ = 22.9 µm.

To characterize the unperturbed optical emission of the TMDC monolayer, we mapped the full PL of the flake. The 532 nm continuous wave (cw) excitation laser is scanned across the WSe_2_ flake, and a time‐integrated PL emission spectrum is recorded at each step (see Experimental Section). The intensity of each spectrum is integrated over an energy range from 1.5 to 1.8 eV and plotted in Figure 1c in a false color map. The shape of the monolayer is clearly resolved in this scan as well as the presence of two ruptures in the monolayer on the bottom left and right of the monolayer (marked by arrows). Figure 1d shows two representative PL spectra at the positions indicated by the colored symbols in Figure 1c (violet cross and dark blue square). In both emission spectra a clear peak is observed which can be attributed to exciton recombination in the WSe_2_ monolayer.^[^ ^3^, ^4^ ^]^ The observed little shift in the emission from both positions can be understood by the impact of the strong dielectric substrate LiNbO_3_ (ɛ_*r*_ ≈ 46) in conjunction with local variation of the dielectric environment caused by inhomogeneities and defects at the WSe_2_‐LiNbO_3_ interface. A more comprehensive analysis of the static PL spectra is available in the Section S1 (Supporting Information).

**Figure 2:**
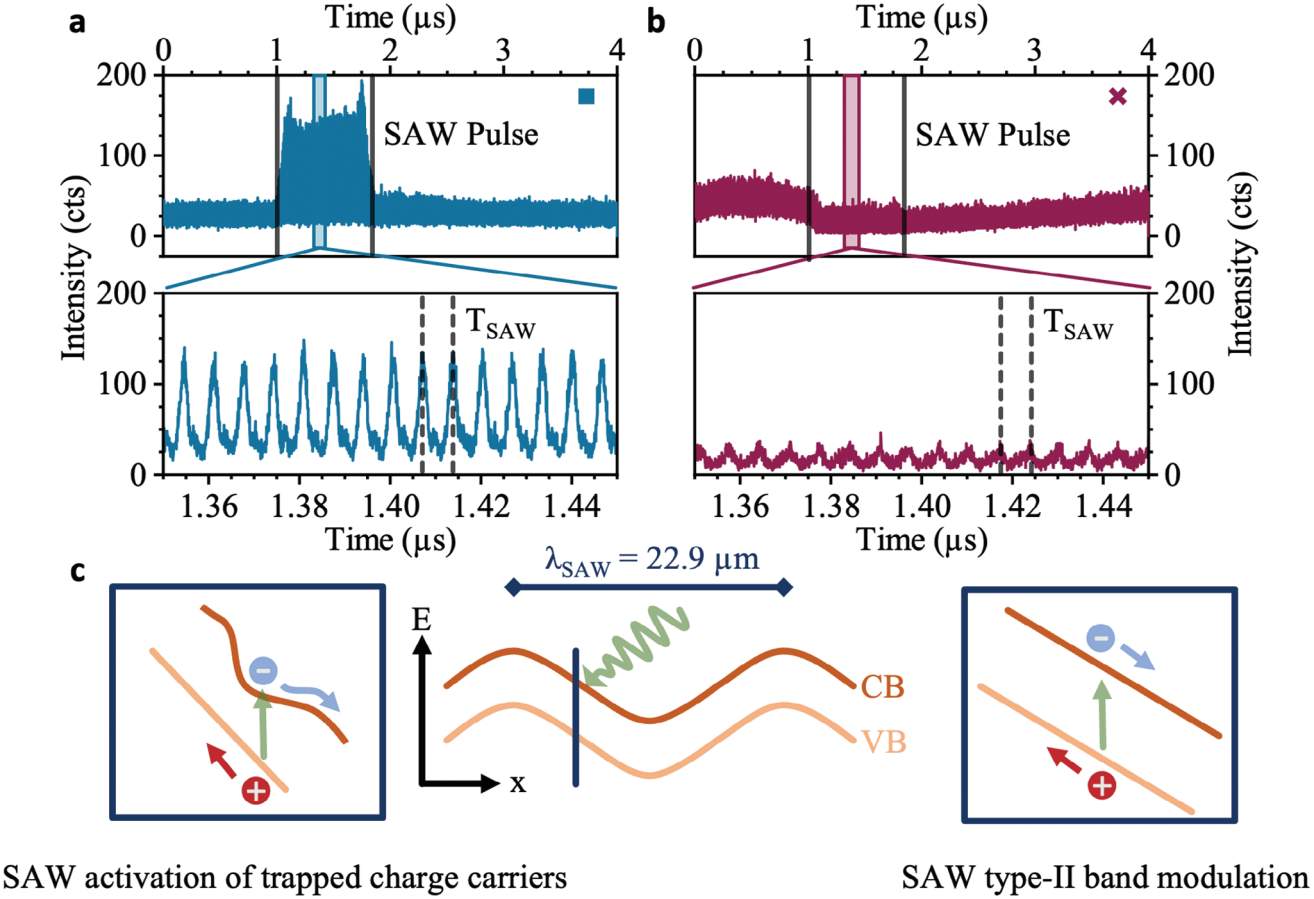
SAW‐modulation of the PL emission of the WSe_2_ monolayer – a,b) Time‐resolved PL emission intensity at two representative positions (symbols corresponding to that in Figure 1c) showing (a) enhancement and (b) suppression of the PL emission during the SAW pulse (upper panels) Center panels: zoom‐ins to time window during the SAW pulse show SAW‐driven modulation of the PL intensity. c) Center panel: schematic of the Type‐II band modulation induced by the SAW piezoelectric field with the dark blue line indicating the position of the light induced scenarios described in left and right panels. Left panel: Poole–Frenkel activation of individual charge carriers from traps by the SAW‐field (here an electron) results in PL enhancement. Right panel: electric field‐driven exciton dissociation leading to PL suppression.

In the following, we address the impact of the *f*_SAW_ = 152 MHz SAW on the WSe_2_ PL emission in Sample 1 and pre‐characterize the induced charge carrier dynamics. For both, Sample 1 and Sample 2, the size of the monolayer is approximately the same as the wavelength of the SAW of λ_SAW_ = 22.9 µm and λ_SAW_ = 10.3 µm, respectively. These size constraints allow to probe the local SAW‐driven carrier dynamics while long‐range transport cannot be studied. Details on the experimental implementation and complementary data from Sample 2 can be found in the Experimental Sections S1–S4 (Supporting Information). To this end, we generate SAW pulses (repetition frequency 250 kHz, on:off duty cycle 1:5) at an electric radio frequency power *P*_rf_ = +31 dBm. **Figure** **2**a,b presents time‐resolved PL intensities under cw laser excitation at the positions presented in Figure 1c,d (dark blue square and violet cross), respectively. The upper panels of Figure 2a,b present data recorded over one pulse repetition period of 4 µs and the lower panels show zoom‐ins to a shorter time interval during which the SAW‐pulse interacts with the WSe_2_ monolayer. We observe clear and very different behaviors at the two positions. These processes are driven by the dynamic electric field of SAW. As shown in Figure 2c the Type‐II bandedge modulation in the WSe_2_ monolayer (center panel). The dark blue line marks one position of maximum electric field amplitude (field pointing along the ‐*x*‐direction) in the SAW cycle.

The observation of very dissimilar behaviors is remarkable because the static PL spectra of these positions (cf. Figure 1d) are very similar and exhibit only a minor energy shift which can be explained by the change in the local dielectric environment due to local differences in the adhesion of the monolayer to the LiNbO_3_ substrate at the two positions.

The data in Figure 2a show a clear ≈ × 20 enhancement of the PL intensity when the SAW pulse interacts with the WSe_2_ in the time interval spanning from 1 to 1.8 µs. Remarkably, this striking effect has not been reported in literature.^[^ ^28^, ^29^, ^30^ ^]^ We attribute this behavior to ubiquitous trapping sites for non‐radiative charge carriers (electrons or holes) which are created by inhomogeneities in the WSe_2_ flake and at the interface to the substrates. The SAW's piezoelectric field activates these trapped charge carriers. Indications of this process have been reported for III–V 2D systems.^[^ ^33^ ^]^ After activation into the continuum in the conduction band (CB) and valance band (VB), excitons form from free electrons and holes. These excitons radiatively recombine giving rise to the observed enhancement of the PL signal. Trap activation processes induced by the applied electric field have been studied extensively for traditional semiconductor structures.^[^ ^34^, ^35^ ^]^ Here, two types of mechanisms have to be considered: the Poole–Frenkel effect,^[^ ^34^ ^]^ i.e., the lowering of the potential barrier of the trap which reduces the activation energy of the initially bound charge carrier, and quantum tunneling effect through the potential barrier.^[^ ^35^ ^]^ Poole–Frenkel activation is depicted schematically in the left panel of Figure 2c for the case of an electron being activated from a trap into the CB continuum.

In contrast, the data in the upper panel Figure 2b exhibits a weak suppression of the PL when the SAW interacts with the WSe_2_ flake. This observation is in qualitative agreement with recent published work^[^ ^28^, ^29^, ^30^ ^]^ as well as reports on established III–V compound semiconductors^[^ ^15^, ^16^, ^18^, ^36^ ^]^ and halide perovskite nanocrystals.^[^ ^21^ ^]^ The observed suppression can be understood qualitatively by a type‐II modulation of the semiconductor band edges by the piezoelectric field of the SAW. This modulation leads to the partial spatial dissociation of the electron and hole composing the exciton and therefore to the suppression of its emission. The process is depicted schematically in the right panel of Figure 2c.

Direct evidence in the emission spectra for both SAW‐induced modulation mechanisms is presented in the center panels of Figure 2a,b. These show zoom‐ins to 100 ns‐long time intervals when the SAW pulse interacts with the photogenerated carriers. Both data sets show clear modulations of the emission intensities with the period of the SAW, *T*_SAW_ = 6.58 ns, the unambiguous fingerprint of SAW‐driven carrier dynamics. Again, the observed modulations exhibit remarkable differences at the two locations. At the position at which we observe a quasi‐stationary enhancement of the PL intensity, the contrast of the modulation is greater than at the position where a suppression is observed.

We probe both phenomena in detail on the full flake. To this end, we scan the full WSe_2_ flake in 1 µm steps and record the time‐resolved PL at each position.

In the first step, we analyze the position dependence of the local enhancement or suppression of the PL signal. To quantify the enhancement, we define the enhancement factor

(1) $$EnF=\frac{I_{ON}-I_{OFF}}{I_{ON}+I_{OFF}}\;$$

**Figure 3:**
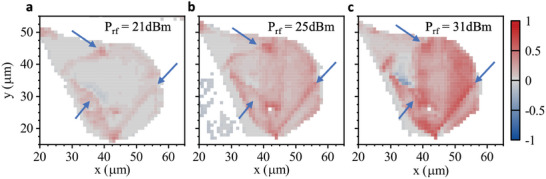
Spatial maps of PL enhancement – False color maps of the enhancement EnF, as a function of the applied radiofrequency power P_rf_ = 21 dBm a), 25 dBm b), and 31 dBm c), respectively. The blue arrows indicate the position of the two cuts in the WSe_2_ monolayer crystal (cf. Figure 1a) and other features with strong PL enhancement.

Here *I*_ON_ and *I*_OFF_ are the root mean square (RMS) emission intensities during the SAW pulse and before the SAW pulse, respectively. Hence, *EnF* > 0 (*EnF* < 0) corresponds to an enhancement (suppression) of the PL during the SAW pulse. Moreover, it approaches ± 1 in the limit of strong enhancement and full suppression. The *EnF* obtained at each point of the scan is plotted in false color maps in **Figure** **3**a–c for three selected rf power levels, *P*_rf_ = 21, 25, and 31 dBm, to the IDT. Data of the full power scan is included in the Supporting Information. At the lowest *P*_rf_ = 21 dBm (Figure 3a), clear features (arrows) become resolvable on the otherwise mostly unaffected PL. These features mark discontinuities and larger scale defects in the monolayer at which, in our interpretation, carrier localization is enhanced. When *P*_rf_ increases (Figure 3b,c), these features become better resolved and an enhancement (red) is observed over almost the full WSe_2_. Contrasting, suppression (blue), which is commonly assumed to be the dominant process, is found only in a small part in the upper left corner of the flake. Our observation is remarkably different from the ones previously reported in the literature.^[^ ^28^, ^29^, ^30^ ^]^ However, these works have key differences, such as the encapsulation of the WSe_2_ monolayer in hBN^[^ ^28^, ^30^ ^]^ or experiments at low temperature on a different TMDC material.^[^ ^29^ ^]^ Thus, our experiment was to study the fundamental interaction between the SAW and the semiconductor monolayer without the presence of an encapsulation layer which could lead to loss of adhesion to the LiNbO_3_ substrate.^[^ ^37^ ^]^ Moreover, our results are robust and reproducible in Sample 2. A summary of the main results of this study is presented in the Section S4 and Figures S4 and S6 (Supporting Information).

As mentioned previously, the trap release behavior influenced by the applied electric field can be described by two regimes: a Poole–Frenkel emission, where a lowering of the potential wall leads to the release of the trapped charge carrier, and a tunneling effect through the potential barrier, which are both enhanced by the electric field.^[^ ^34^, ^35^ ^]^ Poole–Frenkel emission is generally dominant at low electric field amplitudes. In contrast, tunneling activation occurs at large field amplitudes, which in this high field limit effectively lower the barrier triggering carrier escape.^[^ ^35^ ^]^ As shown in the Section S3 (Supporting Information), the in‐plane component of the SAW's electric field in our devices amounts to *F*_*x*,*max*_ ≤ 20 kV cm^−1^. This corresponds to a root‐mean‐square (rms) electric field of *F_rms_* ≤ 13.6 kV cm^−1^. At these moderate fields, Poole–Frenkel activation is expected to dominate, supported by a recent report on polycrystalline monolayer MoS_2_ memtransistor in the low electric field regime.^[^ ^38^ ^]^ Thus, we analyze the corresponding change of the rate at which the trapped charge carriers are activated into the band. For the Poole–Frenkel effect, a decrease of the activation energy Δ*E_i_* leads to an increase of the unmodulated thermal activation rate Γ and is given by

(2) $$\Gamma^{\prime }=\Gamma \cdot \exp\left(\frac{\Delta E_{i}}{k_{B}T}\right)$$

with *T* and *k_B_* being the temperature and the Boltzmann constant, respectively. Assuming a Coulombic trap potential for the defects in our monolayer semiconductor, the change in activation energy Δ*E_i_* as a function of the SAW‐induced *F_rms_* can be expressed as

(3) $$\Delta E_{i}=\frac{q^{3/2}}{{\left(\pi \varepsilon_{r}\varepsilon_{0}\right)}^{1/2}}\;\cdot \sqrt{F_{\mathrm{rms}}}$$

Here, *q* denotes the elementary charge, ɛ_*r*_ and ɛ_0_ the permittivity of the material and vacuum, respectively, and *F_rms_* is the electric field applied to the trap. The PL intensity measured for our sample should be directly proportional to the activation rate Γ given by Equations (2) and (3). Thus, the enhancement factor *EnF* can be expressed as

(4) $$EnF=\frac{\Gamma^{\prime }-\Gamma }{\Gamma^{\prime }+\Gamma }=\frac{1-\exp\left(\frac{\Delta E_{i}}{k_{B}T}\right)}{1+\exp\left(\frac{\Delta E_{i}}{k_{B}T}\right)}$$

By rearranging Equation (4), we obtain:

(5) $$\ln\left(\frac{1+\mathrm{EnF}}{1-\mathrm{EnF}}\right)=\frac{q^{32}}{k_{B}T\cdot {\left(\pi \varepsilon_{r}\varepsilon_{0}\right)}^{12}}\cdot \sqrt{F_{\mathrm{rms}}}$$

**Figure 4:**
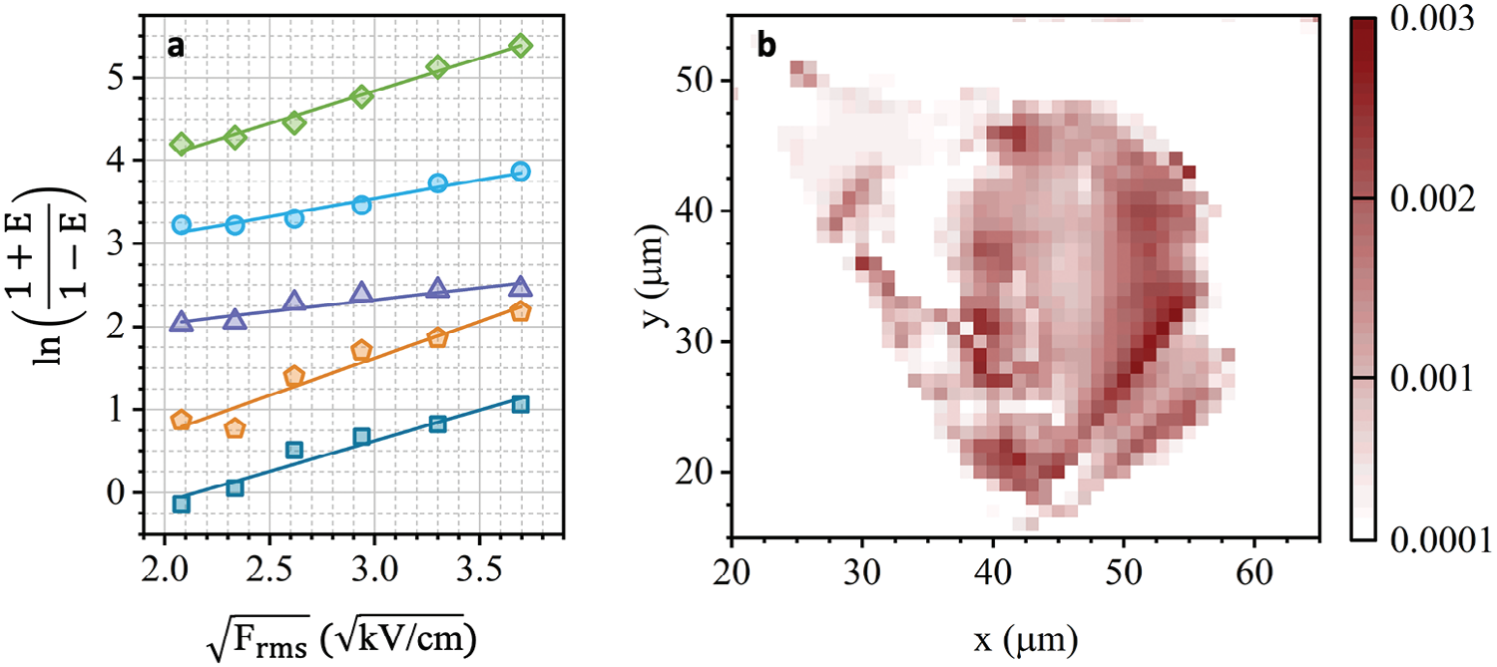
a) Poole–Frenkel activation by SAW electric field– Plot of $\ln(\frac{1+EnF}{1-EnF})$, with EnF the enhancement factor, as a function of $\sqrt{F_{rms}}$, with F_rms_ the root‐mean‐square electric field of the applied SAW, for different positions inside the WSe_2_ monolayer marked in Figure 1c. The data are shifted vertically for clarity and are fitted with a linear fit, which demonstrates a good agreement with the Poole–Frenkel activation model. The measured positions are indicated in Figure 1c with the corresponding symbol. b) False color plot of the slope of the linear fit of $\ln(\frac{1+EnF}{1-EnF})$ as a function of $\sqrt{F_{rms}}$.

Following Equation (5), Poole–Frenkel activation exhibits a characteristic linear dependence of $\ln(\frac{1+EnF}{1-EnF})$ on $\sqrt{F_{rms}}$, with a slope $A=\frac{q^{32}}{k_{B}T\cdot {\left(\pi \varepsilon_{r}\varepsilon_{0}\right)}^{12}}$. Note that Equation (5) is independent on Δ*E_i_* and accounts for the rms change of the activation rate Γ by the SAW. For the following analysis, we calculate $\ln(\frac{1+EnF}{1-EnF})$ from our data. In **Figure** **4**a, we plot $\ln(\frac{1+EnF}{1-EnF})$ extracted at the exemplary positions defined in Figure 1c as a function of $\sqrt{F_{rms}}$, calculated for the different SAW driving power *P_rf_* applied to our IDT (Section S3, Supporting Information). These data are plotted as the same symbols used before in the context of Figure 1c. All data exhibit precisely the anticipated linear dependence which is further corroborated by linear best fits (lines) to the experimental data. Thus, our analysis provides evidence for Poole–Frenkel activation induced by the SAW as the origin of the observed enhancement of the PL intensity. Obviously, the slope of the linear fit *A* is also strongly position‐dependent. Importantly, the only free parameter in this fit is the permittivity. However, the obtained values do not suffice an absolute measure of ɛ_*r*_. The later discussed dynamic modulation of the PL by the SAW leads to an overall decrease of the PL intensity.^[^ ^17^, ^19^, ^39^, ^40^ ^]^ Thus, the value obtained from the fit is an effective permittivity ɛ_*r*,*eff*_, which can approach ɛ_*r*,*eff*_ → 0. As pointed out, ɛ_*r*,*eff*_ cannot be used to determine quantities like the exciton binding energy. We perform the same fitting procedure at each position of our scan at which an enhancement of the PL emission (*EnF* > 0) is observed. The obtained slope *A* is plotted in false color representation as a function of position in Figure 4b. Comparing this plot to the one in Figure 3c, we can see a clear correlation between large enhancement factor, *EnF*, and large slope values, *A*. The extracted value of ɛ_*r*,*eff*_ from our model, ɛ_*r*,*eff*_ being the only free parameter of the fit, ranges from ɛ_*r*,*eff*_ =  1 in strongly enhanced regions to ɛ_*r*,*eff*_ ≈ 15 in the weakly enhanced regions. Values in this range are expected since at lower (upper) interface the monolayer is in proximity to LiNbO_3_ with $\varepsilon_{r}∼46$ (or vacuum). Furthermore, the spatio‐temporal carrier dynamics driven by the SAW, which lead to the observed intensity oscillations, tend to reduce the radiative yield. This effect reduces *EnF* and may result even in physically not meaningful ɛ_*r*,*eff*_ < 0. However, this contribution cannot be faithfully discerned in the data.

**Figure 5:**
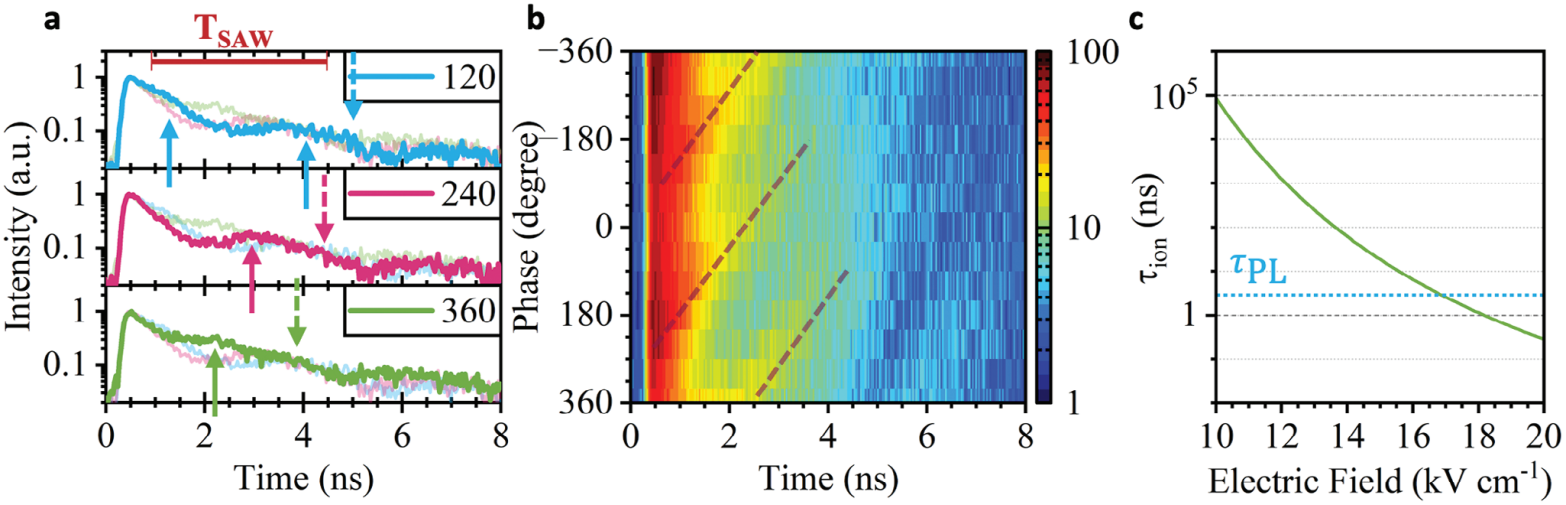
a) Observation of SAW‐driven spatio‐temporal carrier dynamics – Time‐resolved measurement of the exciton PL decay under pulsed excitation of the laser and with a phase difference between the laser and SAW signal of 120°, 240°, and 360°, respectively. The light lines in each panel show the data for the other phase for better comparison. The arrows show the position of higher recombination probability under the SAW‐induced charge carrier dynamics split by T_SAW_ (solid arrows) and T_SAW_/2 (dashed arrows). b) False color map of the time‐resolved measurement of the exciton decay under pulsed laser excitation as a function of the relative phase between the laser and the SAW. The dashed line shows the evolution of the time delayed signals marked in a. The slope of these lines corresponds to the phase velocity of the SAW. c) Calculated exciton dissociation, τ_*ion*_ (solid line) as a function of in‐plane electric field. The dashed line marks τ_*PL*_.

Next, we analyze the time‐dependence of the PL modulation during the SAW pulse which is observed in the data in the lower panels of Figure 2a,b. We first performed fully‐fledged time‐resolved measurements using pulsed laser excitation on Sample 2. The decay time of the PL emission was determined to be τ_*PL*_ =  2.85 ns (see Section S4 and Figure S5, Supporting Information), which is close to the SAW period and at the same time sufficiently long to observe the PL modulation induced by the SAW. We establish a stable phase lock between the laser pulse train and the SAW oscillation by fixing the repetition rate of the laser to $\;f_{laser}=\frac{1}{5}\;f_{\mathrm{SAW}}=67.6\;\mathrm{MHz}$. Thus, charge carriers are generated at a well‐defined local phase of the SAW by controlling the phase difference between the two electrical signal used to excite the SAW and to trigger the laser pulses.^[^ ^18^, ^19^ ^]^ In **Figure** **5**a, we plot the recorded time‐transient for three relative phases between the SAW and the laser pulse. All transients exhibit time‐delayed *T*_SAW_‐periodic enhancements of the PL intensity, marked by upward solid arrows in the figure. The relative phase between the laser pulse and the SAW shifts the time at which these signatures are observed, showing that this recombination happens at a precisely defined local phase of the wave. This observation marks a characteristic and unambiguous fingerprint of SAW‐driven carrier dynamics of the photogenerated carriers. We further analyzed our data in Figure 5b, where we plot the detected PL intensity as a function of time (horizontal axis) and phase difference (vertical axis) over two acoustic cycles. In this plot, the phase shift of the strongest time‐delayed emission (full arrows in Figure 5a) is clearly visible and marked by the dashed lines. The slope of this lines corresponds to the phase velocity of the SAW. The data in Figure 5a contain a second, weaker modulation marked by downward dashed arrows. This modulation is shifted by *T*_SAW_/2 with respect to the primary modulation, which is known to arise from dissimilar mobilities of electrons and holes^[^ ^18^, ^21^ ^]^ or disorder‐induced bandedge modulations.^[^ ^19^ ^]^

To quantify the timescale on which exciton dissociation occurs, we apply a theoretical model derived for 2D semiconductors.^[^ ^41^, ^42^ ^]^ Figure 5c shows the calculated exciton dissociation time given by $\tau_{ion}=\frac{\hbar }{E_{B,X}}\;\frac{\sqrt{2\pi }}{8}\sqrt{\frac{q\cdot F_{x}\cdot a_{0,X}}{E_{B,X}}}\exp\left(\frac{4}{3}\frac{E_{B,X}}{q\cdot F_{x}\cdot a_{0,X}}\right)$, (solid line) as a function of *F_x_*. In this expression, *q* is the elementary electron charge and we assume *E*_*B*,*X*_ =  100 meV and ɛ_*r*_ =  22.5 accounts for the dielectric screening by the LiNbO_3_ substrate.^[^ ^28^ ^]^ Note that ɛ_*r*,*eff*_ determined from the Poole–Frenkel fit cannot be used here as an input parameter since it is an effective quantity and not a quantitative measure for the local permittivity. The Bohr radius of the exciton was calculated to $a_{0,X}=\frac{4\pi \cdot \varepsilon_{0}\varepsilon_{r}\cdot \hbar^{2}}{q^{2}\cdot \mu }=5.15\;\mathrm{nm}$, with µ  =  0.23*m_e_* being the reduced effective mass of the exciton (in units of the free electron mass *m_e_*).^[^ ^43^ ^]^ For efficient exciton dissociation, τ_*ion*_ has to be shorter than the PL decay time τ_*PL*_ (dashed horizontal line). Our model nicely confirms that for realistic parameters, the dissociation time falls below the PL decay time (τ_*ion*_ < τ_*PL*_) for *F_x_* > 17 kV cm^−1^. Thus, we can conclude that exciton dissociation is partially expressed in our sample, and, in general, in any material with similarly high *E*_*B*,*X*_. This conclusion is further supported by numerous reports on III‐V semiconductors^[^ ^16^, ^17^, ^18^, ^39^ ^]^ and recent experiments on halide perovskites.^[^ ^21^ ^]^ In the latter, the excitons binding energy amounts to *E*_*B*,*X*_ =  15 − 25 meV and consequently the observed contrast of the SAW‐driven intensity oscillations is significantly larger than for the WSe_2_ monolayer studied here.

**Figure 6:**
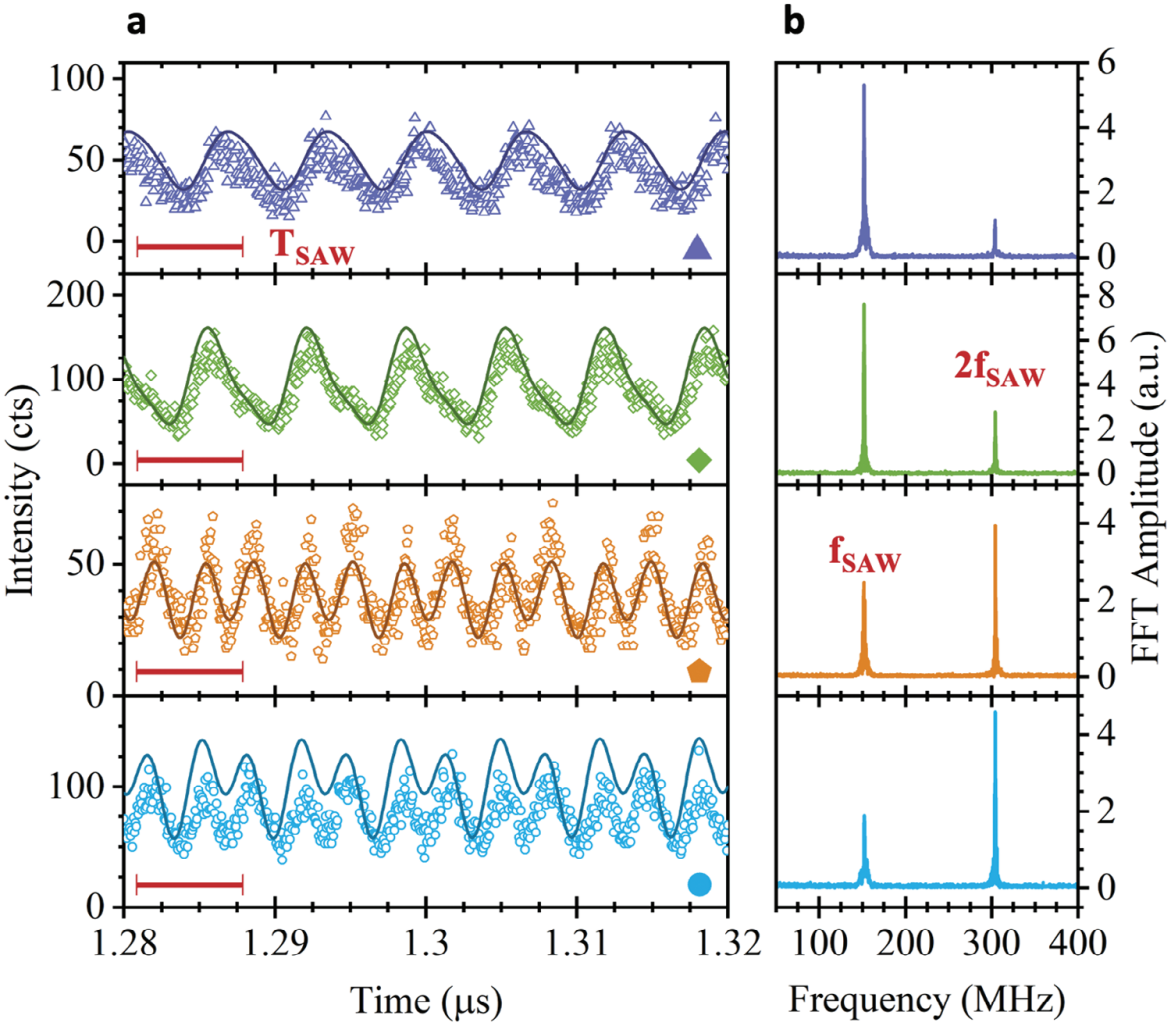
Dynamic PL intensity modulation by SAW under cw laser excitation – a) Time‐resolved modulation of the PL intensity (symbols) by the SAW at four different positions marked in Figure 1c (Scale bar: T_SAW_ = 6.58 ns) Best fit (lines) of Equation (6) to the data. b) Amplitude of FFT of the PL signal over the complete 4 µs time transient showing two peaks at modulation frequencies, f_SAW_ and 2f_SAW_.

As shown in Figure 2, this temporal modulation is clearly resolved even when the material is photoexcited by a cw laser. In contrast to the elaborate phase‐locked excitation scheme under pulsed laser excitation, the cw scheme requires only short integration times (*t*_*int*,*cw*_ < 60 s, *t*_*int*,*pulsed*_ > 120 s). Therefore, fully‐fledged scanning experiments are feasible for our cw excitation scheme and in the following, we present a detailed analysis on the position dependence of the SAW‐driven emission dynamics of the WSe_2_ monolayer. In **Figure** **6**, we study the time‐resolved modulation of the PL emission during the SAW pulse for four representative positions in Sample 1. The symbols correspond to those in Figure 1c and, for reference, we plot the unperturbed PL spectra at these positions in the Supporting Information (cf. Figure S2, Supporting Information). Importantly, we find at different positions on the WSe_2_ flake very distinct types of time‐modulation of the PL signal (symbols). The data recorded in the two upper panels of Figure 6a show a clear domination of *T*_SAW_‐periodic (frequency *f*_SAW_) modulation of the PL in the time‐domain. In contrast, we observe a halving of the period to *T*_SAW_/2 (corresponding to a modulation frequency of 2*f*_SAW_) as the dominating time scale in the data in the lower two panels of Figure 6a. Signature of this *T*_SAW_/2 contribution is also apparent under pulsed excitation in Sample 2 (cf. Figure 5a). These dissimilar dynamics are highlighted looking at the fast Fourier transform (FFT) of the signal over the complete 4 µs range. The FFT amplitudes evaluated at each position are plotted in Figure 6b next to the raw data in the corresponding color. As expected, the two anticipated contributions at *f*_SAW_ and 2*f*_SAW_ are found at all the positions. However, their amplitudes clearly depend on the position at which the measurement was taken and, thus, carry information on the local properties of the monolayer. As noted before, the static PL spectra (Figure S2, Supporting Information) recorded at each position are very similar in line shape and peak energy and do not show direct correlations to the very dissimilar PL modulation dynamics induced by the SAW. This underpins further that the SAW‐modulated PL and its underlying SAW‐induced charge carrier dynamics provide a sensitive local probe of carrier trapping and release from very local perturbations and defects in the 2D semiconductor. For example, the two distinct types of modulation can be observed at the two positions marked by the dark blue square and the orange pentagon in Figure 1c, which are separated by < 2.2 µm and show only minute difference in the static PL spectrum.

The emergence of modulations at *f*_SAW_ and 2*f*_SAW_ depends on two main parameters, the transport mobilities of electrons and holes and band edge variations forming barriers inhibiting SAW‐driven transport. The observed PL modulations at *f*_SAW_ and 2*f*_SAW_ are expected for both pristine semiconductors and in materials in which defects trap carriers or create barriers which inhibit carrier transport by the SAW. This type of modulation is commonly observed in near‐pristine III‐V quantum wells and nanowires^[^ ^17^, ^18^, ^40^, ^44^ ^]^ in which spatio‐temporal carrier dynamics occur within the Type‐II modulated band structure (cf. Figure 2c). There, the observed modulation directly reflects the mobilities of the charge carriers. In most semiconductors, in particular III‐V compounds like (Al)GaAs, the mobility of one carrier species, typically the electron, is greater than that of the other species, typically the hole.^[^ ^18^ ^]^ In this scenario, the lower mobility carrier species remains quasi‐stationary at intermediate SAW‐amplitudes, and this leads to a *T*_SAW_‐periodic modulation. The second case, where the mobilities of both carrier species are comparable and both carrier species are mobile, i.e., no trapping occurs, is also present in pristine materials. The opposite charges of the two carrier species lead to anti‐phased dynamics and a spatial overlap twice per acoustic cycle and to a *T*_SAW_/2‐periodic PL‐modulation. The corresponding dominant frequency component 2*f*_SAW_ has been reported for CsPbI_3_ nanowires and provided the first direct evidence of the predicted near‐identical mobilities of electrons and holes in this class of materials.^[^ ^21^ ^]^ Detailed schematics for these SAW‐induced charge carrier dynamics are available in the Supporting Information.

In our sample, and 2D materials in general, the unmodulated band structure cannot be assumed to be as spatially homogeneous like in epitaxial III‐V quantum wells. On the contrary, local defects and inhomogeneities create random modulations of the band edges and trap carriers. Recent work showed that, for example, trapping of carriers in crystal phase mixing^[^ ^18^, ^19^ ^]^ or thickness fluctuations in tubular quantum wells^[^ ^45^, ^46^ ^]^ in III‐V nanowires leave pronounced fingerprints in the AOES data. There, an inhomogeneous distribution of trapping sites creates both *f*_SAW_ and 2*f*_SAW_ components which overlap in the time‐resolved measurements.^[^ ^19^ ^]^ In our data, we observe that both types of modulations can be superimposed and variations of both contributions occur on short length scales of a few µm. This, and considering further the discussed enhancement of the mean PL intensity, strongly suggest that the SAW‐driven carrier dynamics in 2D materials cannot be explained by the simple model assuming a near pristine system which has been commonly assumed.^[^ ^28^, ^30^ ^]^ Thus, our observations have to be understood on the basis of static perturbations created by defects and inhomogeneities. Examples of such a charge carrier dynamic are schematically detailed in the Figure S7 (Supporting Information).

**Figure 7:**
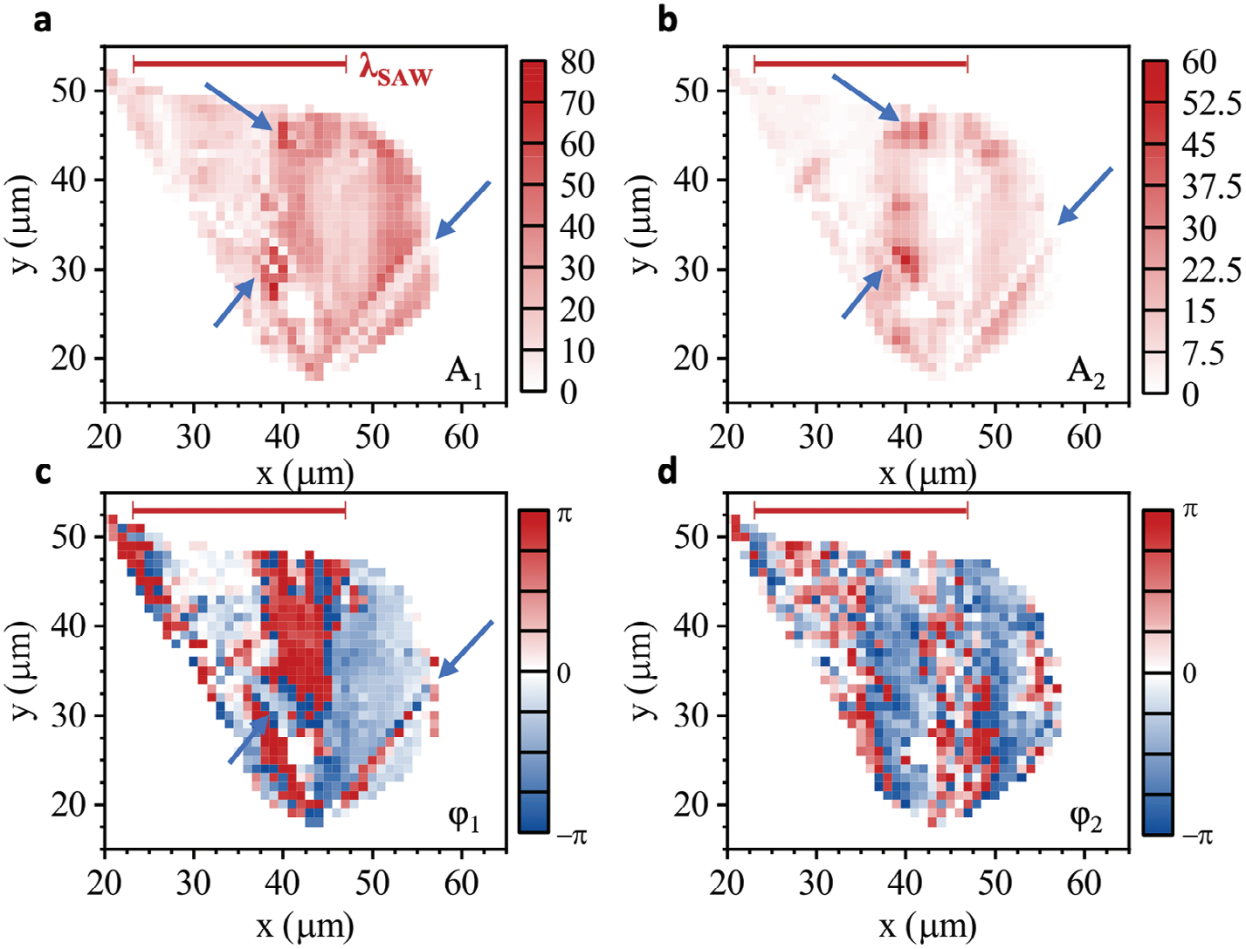
Analysis of frequency contributions – False color map of obtained fit parameters: Amplitude, phase of f_SAW_. a,c) and and 2f_SAW_ b,d) contribution, Scale bar: λ_SAW_ = 22.9 µm.

To this end, we quantify the two contributions to the modulation and fit a superposition of two sinusoidal modulations of frequencies *f*_SAW_ and 2*f*_SAW_ to the time‐resolved PL‐signals. We perform this analysis at each point of the scan. The corresponding fit equation is given by:

(6) $$I\;\left(t\right)=I_{0}+A_{1}\cdot \sin\left(2\pi \cdot f_{\mathrm{SAW}}\cdot t+\varphi_{1}\right)+A_{2}\cdot \sin\left(2\pi \cdot \left[2f_{\mathrm{SAW}}\right]\cdot t+\varphi_{2}\right)$$

where *A*_1_ and *A*_2_ are the amplitude and *φ*_1_ and *φ*_2_ the phase of the *f*_SAW_ and 2*f*_SAW_ contributions, respectively. The results of these best fits of Equation (6) are plotted as lines in Figure 6a. From these fits at every point of the scan and *P*_rf_, we extract *f*_*SAW*,*fit*_ =  152 MHz and the four free parameters *A*_1_, *A*_2_, φ_1_, and φ_2_. **Figure** **7** shows *A*_1_ (a), *A*_2_ (b), *φ*_1_ (c), and *φ*_2_ (d) extracted at every point of the scan of the flake for an applied power *P*_rf_ = +31dBm.

In these data, the amplitudes *A*_1_ and *A*_2_ (Figure 7a,b) are clearly resolved across the full flake and provide evidence that the SAW induces spatio‐temporal carrier dynamics on the full flake. The amplitudes *A*_1_ and *A*_2_ show dissimilar spatial distributions. While *A*_1_ is large over a large portion of the flake, *A*_2_ is detectable in small regions. Most importantly, defects and trapping sites are clearly resolved in the data. Prominent examples like a line defect or crack and local traps are marked by arrows in the plots. The amplitude data are further corroborated by the data of the local phases *φ*_1_ and *φ*_2_ which are presented in Figure 7c,d, respectively. In these data, the corresponding periodicities, *λ*_SAW_ (scalebar) and *λ*_SAW_/2 are clearly resolved as a vertical stripe pattern along the *y*‐direction (perpendicular to the SAW propagation) for *f*_SAW_ and 2*f*_SAW_, respectively. This evidences that the carrier dynamics are faithfully locked to the local acoustic phase and the observed modulations are not induced by electromagnetic crosstalk. Moreover, these data also resolve defects. For example, the line defects lead to constant phase contours which are not aligned in the *y*‐direction, perpendicular to the SAW propagation direction. Additionally, the correlation between the large amplitude of the modulation in region with large enhancement and low amplitude in region with low enhancement and even suppression confirms our theory for the low probability of complete dissociation of the excitons in our sample. The same analysis was also performed by extracting the amplitudes of the *f*_SAW_ and 2*f*_SAW_ contributions from the FFT of the signal. The data for this analysis is available in the Supporting Information (cf. Figure S8, Supporting Information) and leads to the same conclusions.

In the next step, we quantify the relative contributions of the *f*_SAW_ and 2*f*_SAW_ modulations by defining the degree of modulation given by

(7) $$M=\frac{A_{2}-A_{1}}{A_{1}+A_{2}}$$

**Figure 8:**
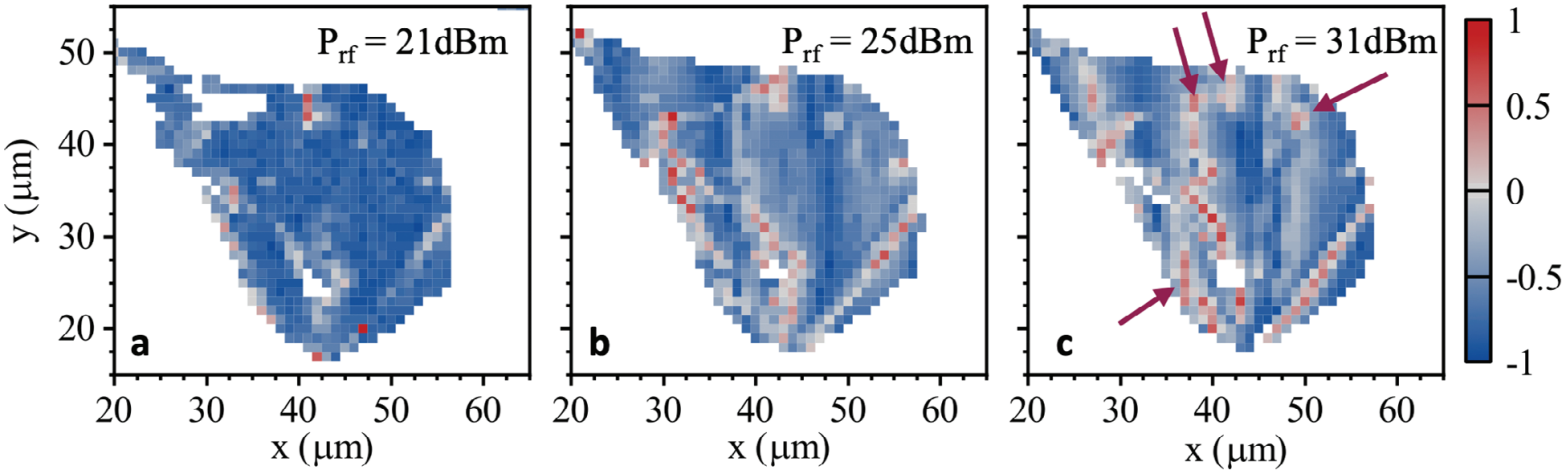
Weight of *f*_SAW_ and 2*f*_SAW_ contributions – False color maps of M for P_rf_ = 21 dBm a), 25 dBm b), and 31 dBm c), respectively. Arrows mark the positions of local trapping sites not resolved in conventional scanning PL.

Here, *M*  =  0 for both modulations having equal amplitudes and approach *M*  =   ± 1 for the cases where either 2*f*_SAW_ or *f*_SAW_ dominate strongly. Spatial maps of *M* for *P*_rf_ = 21, 25, and 31 dBm (see Supporting Information for a full power series) are plotted in false color representation in **Figure** **8**a–c. At a moderate SAW amplitude (*P*_rf_ = 21 dBm, Figure 8a), the relative contribution is dominated by *M*  =   − 1 (blue) over almost the entire area of the flake. This *f*_SAW_‐modulation is expected for a regime in which the finite mobilities limit the dynamics.^[^ ^17^, ^19^, ^40^ ^]^ As the SAW amplitude increases to *P*_rf_ = 25 dBm (Figure 8b) and *P*_rf_ = 31 dBm (Figure 8c), 2*f*_SAW_‐modulation (*M*  =   + 1, red) appears. This modulation does not emerge over the entire flake, as it would be expected for a homogeneous, near pristine material.^[^ ^17^, ^40^ ^]^ In contrast, it is observed only locally pointing toward a microscopic origin. Section S5 (Supporting Information) presents possible scenarios and the expected time‐modulations. For instance, when *P*_rf_ increases, spatial confinement of one carrier species still occurs and the other carrier species is already driven toward and away from that site twice per acoustic cycle. This induces a 2*f*_SAW_‐periodic modulation of the signal. Most strikingly, the 2*f*_SAW_ modulation resolve local line defects and local barriers (arrows) which are not visible in the conventional static PL scan (cf. Section S1, Supporting Information). This nicely demonstrates the high sensitivity and high spectral resolution of our scanning acousto optoelectronic spectroscopy.

## 3 Conclusion

In conclusion, we systematically investigated the SAW‐induced charge carrier dynamics in a monolayer of WSe_2_ transferred onto LiNbO_3_. We observed a clear enhancement of the average PL signal when the SAW interacts with the 2D semiconductor. This effect is found to be in excellent agreement with Poole–Frenkel activation of defects in the material by the dynamic electric field of the SAW. Furthermore, we presented direct evidence of SAW‐driven carrier dynamics, the temporal modulation of the PL emission. We observe a characteristic, *T*_SAW_‐periodic modulations of the PL signal. These modulations set in when the exciton dissociation time falls below the PL decay time exceeds, as corroborated by theoretical modelling using realistic parameters. We find that the observed modulation contains two frequency contributions at *f*_SAW_ and 2*f*_SAW_. This indicates that these modulations cannot be explained solely by the commonly assumed spatio‐temporal carrier dynamics in the Type‐II band edge modulation induced by the piezoelectric SAW. Hence, these contributions arise from a combination of the Type‐II band edge modulation and a static disorder potential induced by inhomogeneities and defects in the monolayer semiconductor. We introduce two figures of merit *M* (relative contributions of *f*_SAW_ and 2*f*_SAW_ modulation) and *EnF* (degree of SAW‐driven PL enhancement) which quantify both effects. In spatial maps, both figures of merit faithfully resolve local defects and trapping sites. Most importantly, these features are not resolved in conventional scanning static PL experiments. This validates our method as a powerful and fully‐fledged contact‐free spectroscopic technique which can be applied to a wide range of emerging low‐dimensional materials. Examples include optically active 2D semiconductors beyond WSe_2_ in this work, van der Waals stacks^[^ ^31^ ^]^, organic semiconductors^[^ ^47^ ^]^ or hybrid perovskites.^[^ ^21^, ^48^ ^]^ Finally, note that our method can be combined with direct SAW‐spectroscopy and electrical transport measurements to map optical data onto the electric domain.^[^ ^13^ ^]^ This will provide invaluable insights for the optimization of hybrid acousto‐optoelectric devices.^[^ ^10^, ^13^, ^27^, ^49^, ^50^ ^]^

## 4 Experimental Section

### Sample Fabrication and Characterization

The SAW devices were fabricated on Y‐cut LiNbO_3_, using a standard optical lithography and lift‐off process and the rf scattering parameter S_11_ of the IDT was determined using vector network analysis. The IDT designs of Sample 1 and Sample 2 facilitate generation of *f*_SAW_ =  152 MHz (λ_*SAW*_  =  22.9 µm) and *f*_SAW_ =  338 MHz (λ_*SAW*_  =  10.3 µm), respectively. A monolayer of WSe_2_ was mechanically exfoliated from a bulk crystal and transferred onto the targeted substrate using a dry transfer technique utilizing a PDMS stamp.

### Acousto‐Optoelectronic Spectroscopy

All PL experiments were performed at room temperature (*T* = 300 K) with the sample in vacuum (*p* < 10^−5^ mbar). These measurements were performed using a 532 nm laser (time averaged power 100 µW in cw mode and 300 µW in pulsed mode, pulse duration 90 ps) focused to a diffraction‐limited spot (diameter 1 µm) by a 50x (NA 0.65) objective and scanned using a piezo positioning stage. The emitted PL was collected by the same objective lens and analyzed using a 0.5 m grating spectrometer. Multi‐channel detection was performed using a thermoelectrically cooled Si charge coupled device (CCD) detector. For the time‐resolved measurements, the PL signal was collected directly by a multimode optical fiber after being spectrally filtered by an optical long pass 532 nm‐Raman filter and detected by a single photon avalanche diode (timing jitter 300 ps).

The SAW was excited by applying a pulsed rf electrical signal to the IDT. The SAW was synchronized and actively phase‐locked to a time‐correlated single photon counting (TCSPC) module.^[^ ^18^, ^44^ ^]^

## Conflict of Interest

The authors declare no conflict of interest.

## Supporting information



Supporting Information

## Data Availability

The data that support the findings of this study are available from the corresponding author upon reasonable request.
