# Supplementary material for: Scanning Acousto‐Optoelectric Spectroscopy on a Transition Metal Dichalcogenide Monolayer
Source: Adv Mater. 2024 Oct 24;36(49):2402799. doi: 10.1002/adma.202402799 (PMC11619229; doi:10.1002/adma.202402799)
Supplement: Supplementary file 1 — Supporting Information [file ADMA-36-2402799-s001.pdf]

ADVANCED MATERIALS

Supporting Information

for *Adv. Mater.*, DOI 10.1002/adma.202402799

Scanning Acousto-Optoelectric Spectroscopy on a Transition Metal Dichalcogenide Monolayer

Emeline D. S. Nysten, Matthias Weiß, Benjamin Mayer, Tobias M. Petzak, Ursula Wurstbauer and Hubert J. Krenner*

Supporting Information

Scanning Acousto-Optoelectric Spectroscopy on a Transition Metal Dichalcogenide Monolayer

Emeline D. S. Nysten, Matthias Weiß, Benjamin Mayer, Tobias M. Petzak, Ursula Wurstbauer, Hubert J. Krenner*

1. Photoluminescence scans analysis Sample 1:

For static photoluminescence (PL) characterization of the WSe_2 flake, a continuous wave (cw) excitation laser is scanned across the WSe_2 flake by a closed-looped high resolution piezo stage, and a time-integrated PL emission spectrum is recorded at each step. Each of these spectra is fitted by two Gaussian curves (index l =1,2), as depicted in Figure S 1a. From each of these curves, the center energy E_l was extracted and its deviation from the average center energy $\overline{E_l}$ with respect to the full width at half maximum (FWHM) calculated. This difference $\frac{E_l-\overline{E_l}}{\mathrm{FWHM}}$ is plotted in a false color plot for both curves in Figure S 1b and c, respectively. When comparing these plots to the ones in Figure 3, Figure 4, Figure 7, and Figure 8 of the main text, it is clear that the charge carrier dynamics induced by the SAW resolve more details than in these conventional PL measurement. Selected static photoluminescence spectra corresponding to the time-resolved measurements in Figure 6 and the positions indicated in Figure 1c are plotted in Figure S 2.

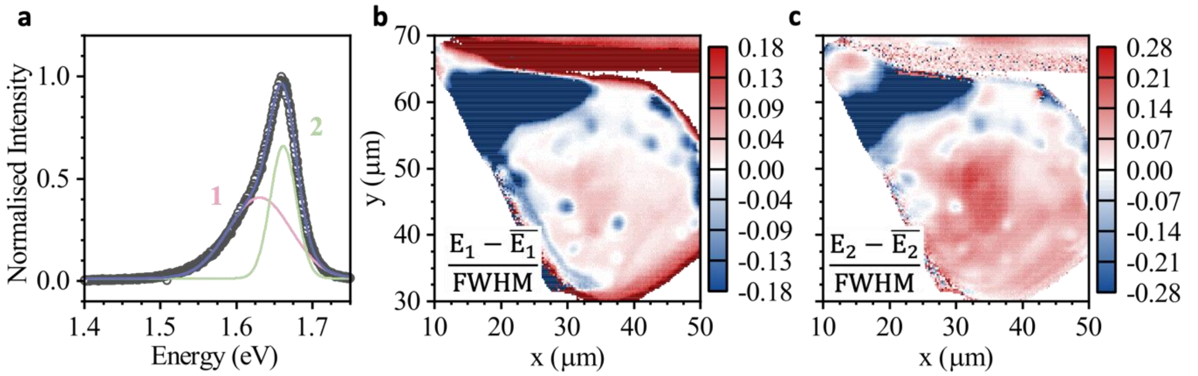

Figure S 1 – Static PL spectral characterization of Sample 1 – **a** *Fit of a typical photoluminescence spectrum of the WSe_2 monolayer with two Gaussian curves.* **b-c** *False color maps of the deviation of the center energy for the fitted gaussian curve compared to the average center energy of all the fitted curve in per cent:* **b** *for the first gaussian E_1 and* **c** *for the second gaussian E_2.*

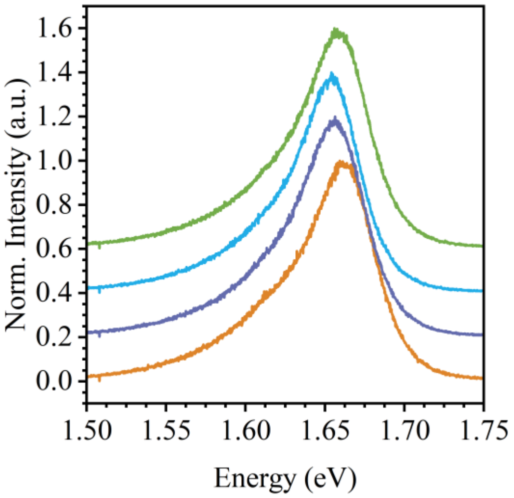

Figure S 2 – Static PL characterization of Sample 1 – PL spectra of the exciton emission in WSe_2 for the positions indicated in Figure 1c and the time-resolved measurement in Figure 6.

2. Enhancement as a function of P_{rf}:

The enhancement factor $EnF = \frac{I_{ON}-I_{OFF}}{I_{ON}+I_{OFF}}$ is plotted in false color maps for the complete range of P_{rf} in Figure S 3. Like stated in the main text, the enhancement of the monolayer PL emission gets stronger as P_{rf} increases, except for a small region in the top left corner of the flake. The enhancement is especially pronounced at defects for each power.

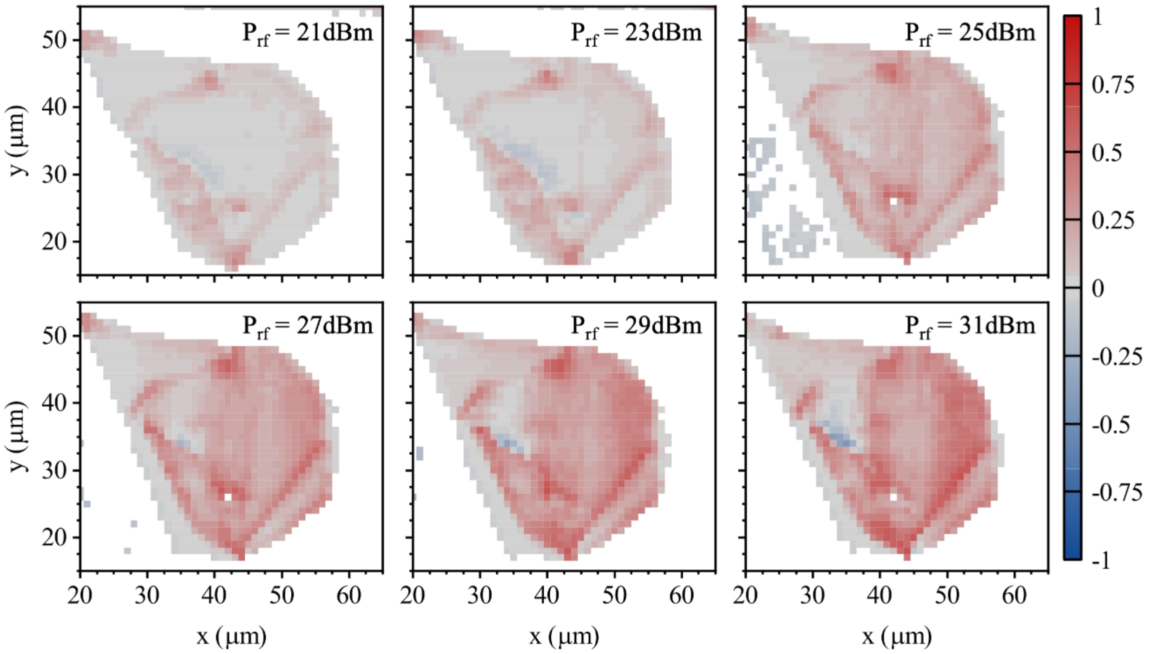

Figure S 3 – PL enhancement maps of Sample 1 – False color maps of the enhancement of the PL emission, given by EnF, as a function of the applied radio frequency power P_{rf} from 21 dBm to 31 dBm in 2 dB steps.

3. SAW electric field calculation:

By using the insertion loss of our delay line of -dB, we can extract the SAW power emitted by our IDT if no additional losses are present. From this, the piezoelectric field of the wave can be

calculated using finite element simulations. Table S 1 shows the different values obtained for our experiments.

P_{in} (dBm)	P_{SAW} (mW)	V (V)	F_z (kVcm^{-1})	F_x (kV cm^{-1})	F_{rms} (kV cm^{-1})
31	629	19.7	19.3	19.5	13.6
29	397	15.7	15.4	15.5	10.9
27	251	12.4	12.2	12.3	8.63
25	158	9.89	9.69	9.76	6.86
23	99.8	7.85	7.7	7.75	5.44
21	62.9	6.24	6.11	6.16	4.32

Table S 1 Calculated electric field amplitude (peak-to-peak and rms) as a function of the applied rf power to the IDT.

4. Analysis of Sample 2

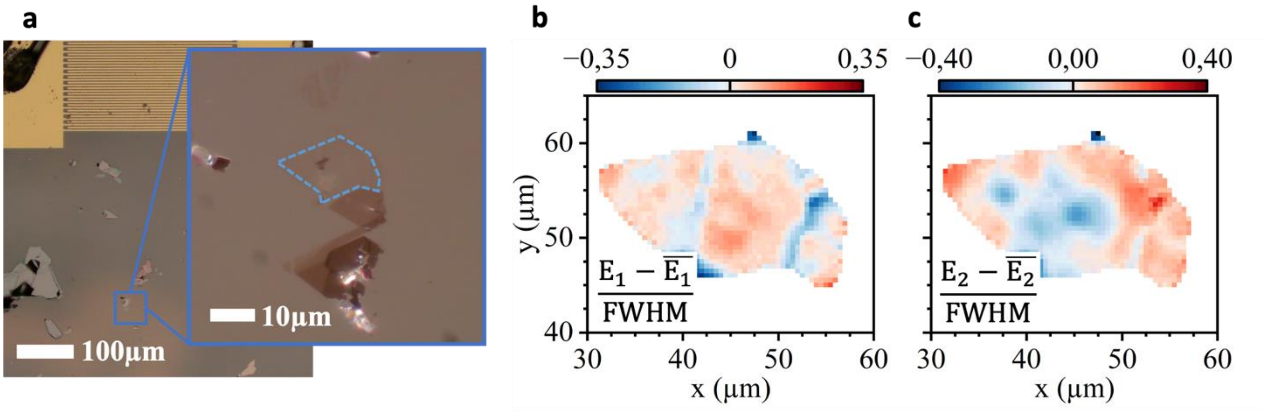

Figure S 4 – Static PL analysis of Sample 2 – **a** *Microscope image of Sample 2. The WSe_2 monolayer is located below the IDT exciting the SAW.* **b-c** *False color maps of the deviation of the center energy for the fitted gaussian curve compared to the average center energy of all the fitted curve in per cent:* **b** *for the first Gaussian E_1 and* **c** *for the second Gaussian E_2.*

In addition to Sample 1, another monolayer of WSe_2 placed inside a SAW delay line on another $LiNbO_3$ substrate, Sample 2. A microscope image of the WSe_2 monolayer is presented in Figure S 4a. For the static photoluminescence (PL) characterization of the WSe_2 flake, a continuous wave (cw) excitation laser is scanned across the WSe_2 flake by a closed-looped high resolution piezo stage, and a time-integrated PL emission spectrum is recorded at each step. Each of these spectra is fitted by two Gaussians, analogous to Sample 1 (cf. Figure S 1a). From each of these curves, the center energy E_l was extracted and its deviation from the average center energy $\overline{E_l}$ with respect to the full width at half maximum (FWHM) calculated. This difference $\frac{E_l - \overline{E_l}}{\mathrm{FWHM}}$ is plotted in a false color plot for both curves in Figure S 4b and c, respectively.

To study the SAW-induced charge carrier dynamics, Sample 1 was first investigated using a pulsed laser spectroscopy to determine the time decay of the PL signal of the monolayer with and without SAW and then in the same manner as Sample 1 with the laser in a continuous wave mode (see Method section for more details). The photoluminescence decay of the WSe_2 monolayer emission under pulsed laser excitation is plotted in Figure S 5 as well as the instrument response function of the laser and the fit of the convolution function between the IRF and the exponential decay of the PL emission used to determine the decay rate of the excitons, $\tau = 2.85$ns. In the right panel of Figure S 5, the PL decay with the influence of the

SAW is plotted alongside the unperturbed decay. The SAW tends to accelerate the decay rate and shows features of the SAW-induced charge carrier dynamics discussed in the main text.

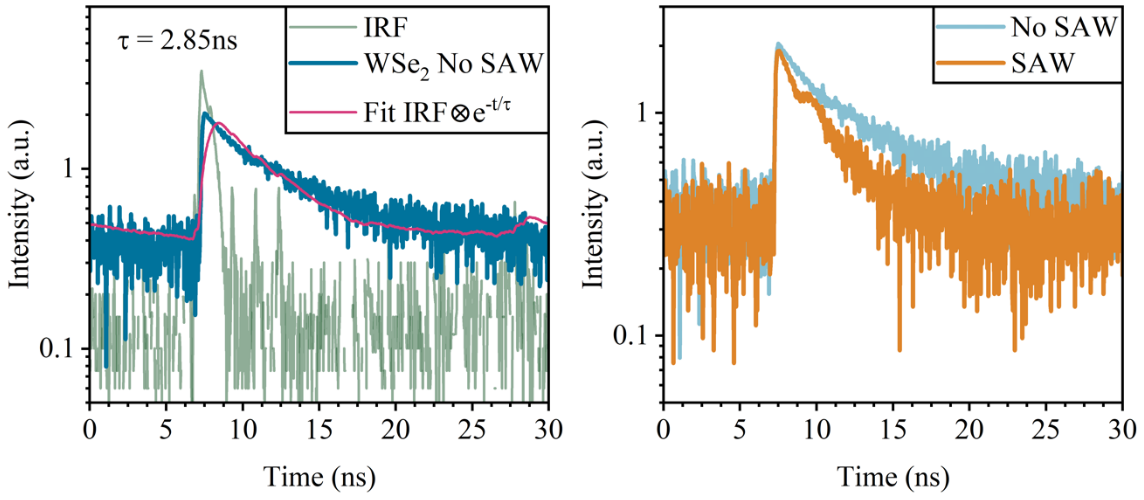

Figure S 5 – PL transients of Sample 2 under pulsed laser excitation – Left panel: Photoluminescence decay of the WSe_2 monolayer emission. The instrument response function (IRF) of the laser is plotted along with the fit of the convolution function between the IRF and the exponential decay of the PL emission. Right panel: Photoluminescence decay of the WSe_2 monolayer emission with and without the SAW-induced modulation of the charge carriers.

The same experiments and analysis presented in the main text were conducted on Sample 2. The photon counts without the SAW are plotted in a false color map in Figure S 6a. The monolayer is clearly visible with the presence of two possible cracks or folds. The SAW, f_{SAW} = 338 MHz, is excited in a pulse of 300 ms at a frequency of 500 kHz with P_{SAW} = +27 dBm. Just like for Sample 1, the enhancement of the PL is evident is the time-resolved measurement plotted for different two positions within the flake in Figure S 6d. The enhancement ratio, $E = \frac{I_{ON}-I_{OFF}}{I_{ON}+I_{OFF}}$, is plotted in a false color map in Figure S 6b and shows a predominance of the SAW-induced enhancement of the PL emission except in a small region at the bottom of the monolayer. The SAW periodic modulation of the PL intensity is also evident in the zoom-in during the SAW pulse in Figure S 6e and the fast Fourier transform of the signal in Figure S 6f shows the presence of two contributions, T_{SAW} and $T_{\mathrm{SAW}}/2$, for the first position (green star in a) and only a T_{SAW} contribution for the second (red star in Figure S 6a), respectively. The ratio between the two harmonics amplitudes, $M = \frac{A_2-A_1}{A_1+A_2}$, is plotted in a false color map in Figure S 6c and shows that, in the case of this monolayer, the T_{SAW} modulation is dominant, except in a small region in the crack of fold on the left of the monolayer.

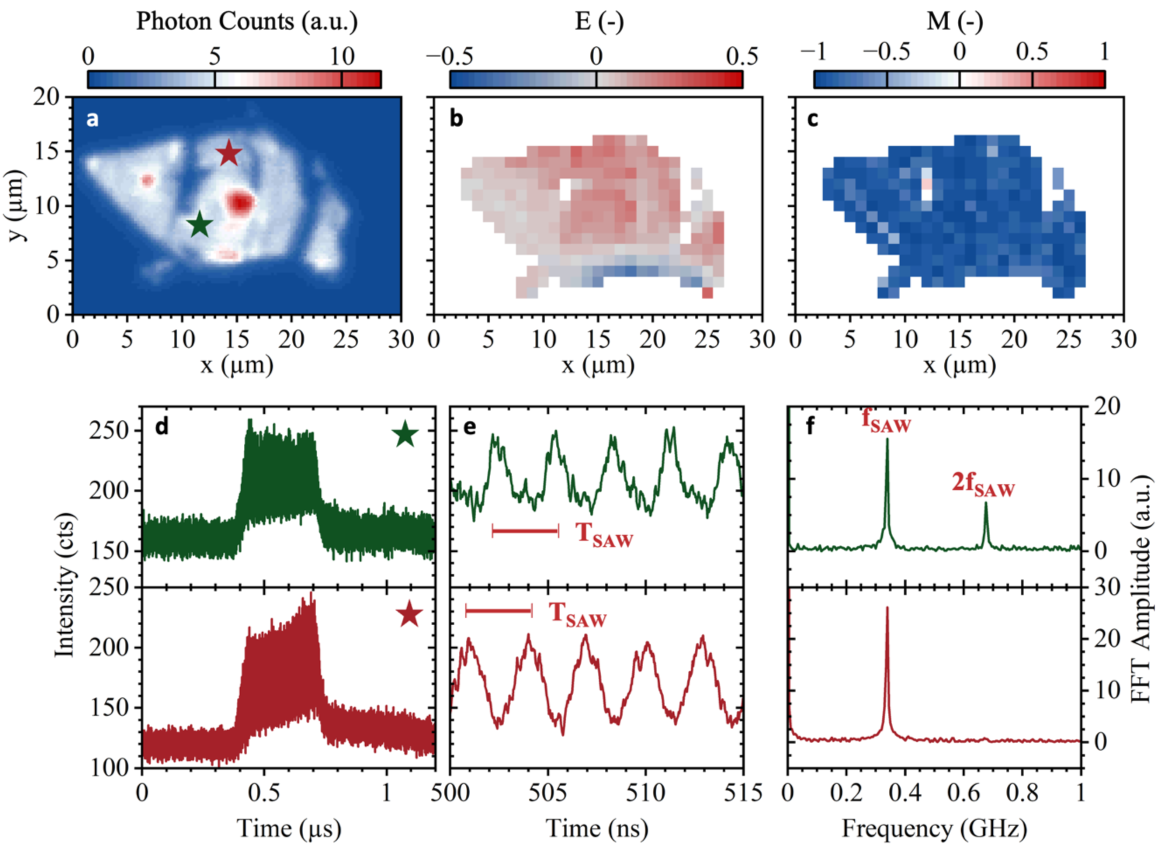

Figure S 6 – Summary of the time-resolved investigation of the SAW-induced charge carrier dynamics in Sample 2 – **a** *False color map of the photon counts number (arbitrary units, a. u).* **b** *False color map of the enhancement E of the PL emission.* **c** *False color map of M, the ratio between the two frequency contributions.* **d** *Time-resolved PL emission intensity at two representative positions (symbols corresponding to that in a) showing an enhancement of the PL emission during the SAW pulse.* **e** *Zoom-ins to a time window during the SAW pulse which show the SAW-driven modulation of the PL intensity.* **f** *Amplitude of the FFT of the SAW modulation of the PL intensity showing both modulation frequencies for the upper case and only the f_{SAW} modulation in the lower case, respectively.*

5. Charge carrier dynamics:

In Figure S 7, we present schematics of exemplary charge carrier dynamics at specific times during the SAW cycle, separated by $T_{SAW}/4$, for three different cases. These and other cases have been studied in detail in previous work using a 1D model.[17–21,40] Our data is fully consistent with earlier works which demonstrate that different local variations electronic properties of the material or different charge carrier mobilities determine the observed PL-dynamics. For a complete overview, we refer the reader to above references and restrict ourselves here to three examples.

In the first case, Figure S 7a, the mobilities of the charge carriers are very dissimilar, in this example the electron is much more mobile than the hole. This scenario corresponds to the system studied in [40] for the cases of intermediate SAW fields. As the SAW amplitude, i.e. electric field is changed the weight of the two frequency contributions change as we observe it also in the data discussed here. The exciton is photogenerated at $t = 0$, when the bands are flat. At $t = 0.25T_{SAW}$, the electric field of the SAW is at its maximum and leads the electron away from the hole up to the position of equilibrium with the exciton bonding energy at $t = 0.5T_{SAW}$. The probability of recombination is then strongly suppressed by the SAW. As the SAW cycle continues, the electric field changes direction and leads the electron back to the hole position.

There, the charge carriers can then recombine. In this case, the modulation of the PL will thus show a T_{SAW} periodicity. The case of similar mobilities for both holes and electrons in Figure S 7b, which corresponds to the scenario in [21]. Here, the SAW cycle leads to higher recombination rates two times per cycle as the carriers move away and then towards each other twice per cycle. Therefore, exclusively a $T_{SAW}/2$ ($2f_{SAW}$) modulation is observed in the PL emission dynamics and the T_{SAW} (f_{SAW}) contribution is completely suppressed.
A third case is shown in Figure S 7c which leads to a $2f_{SAW}$ component in the data due to the presence of defect-induced energy barriers. This scenario was comprehensively investigated in [19]. Again, one carrier (here the hole, red) is quasi-stationary e.g. due to a lower mobility compared to the other carrier species. This other carrier species (here electrons, blue) is transported towards and away from the position of the hole by the oscillating electric field of the SAW. Due to the presence of inhomogeneities, these charge carriers can be reflected by energy barriers on their path, leading to a recombination twice per cycle and, thus, a $2f_{SAW}$ component. Furthermore, the presence of defects can lead to additional photogenerated electrons released from shallow traps by the SAW. These spatially separated electrons have different arrival times which differ by $T_{SAW}/2$, creating the observed $2\,f_{SAW}$ component.

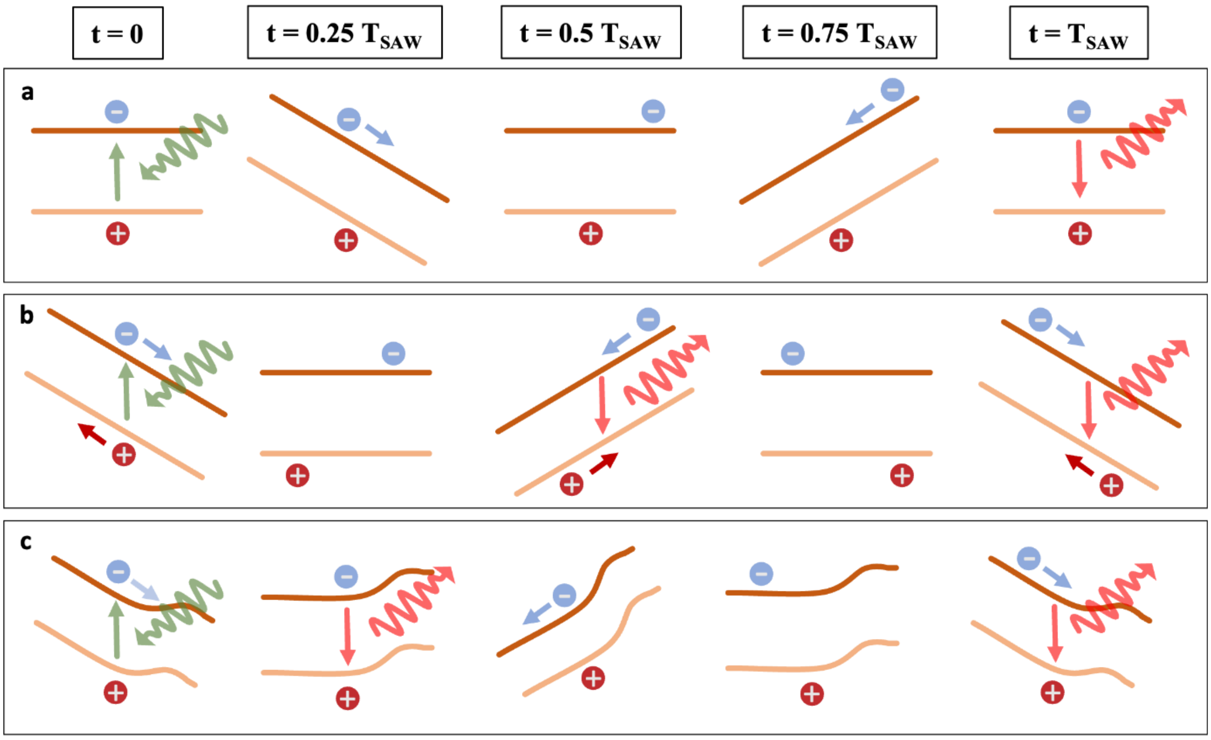

Figure S 7 – Spatio-temporal carrier dynamics – Schematics showing the charge carrier dynamics at selected points during a SAW period for ***a*** *low mobility of holes,* ***b*** *similar mobility for both charge carriers and* ***c*** *for a low hole mobility and the presence of an energy barrier due to, for example, the presence of a potential barrier.*

6. Fast Fourier transform analysis:

The Fast Fourier transform of the time-resolved PL measurement was performed for all position in the WSe_2 flake of Sample 1. The amplitude of the peaks localized at f_{SAW} and $2f_{SAW}$ (as seen in Figure 6b) is extracted and plotted in false color in Figure S 8. The plots are similar and allows the same conclusions as the ones drawn from Figure 7. The modulation ratio $M = \frac{(A_2 - A_1)}{(A_2 + A_1)}$ is plotted in Figure S 8c and is identical to the one obtained by the sinusoidal fit in Figure S 9 and Figure 8 for the same P_{rf}.

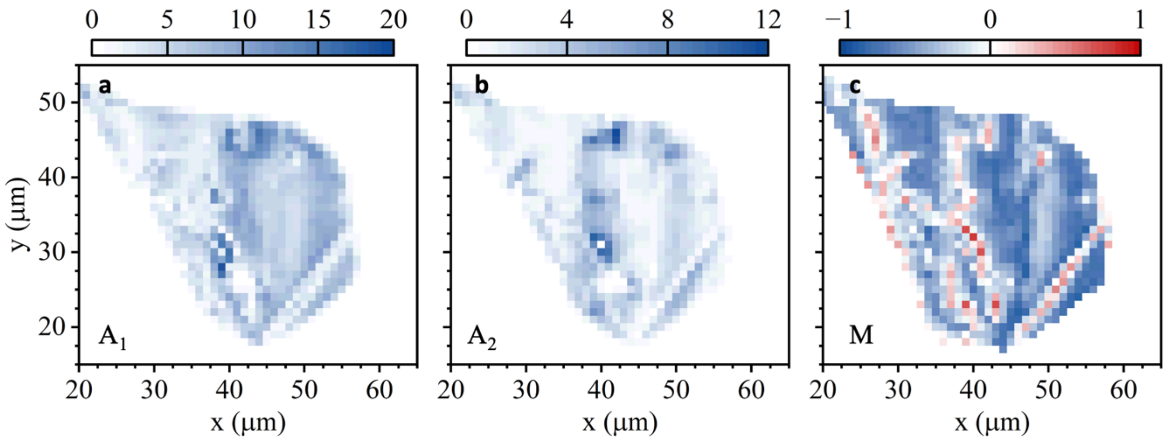

Figure S 8 – Frequency domain analysis –FFT of the time-resolved PL transients of the WSe_2 monolayer. **a-b** *False color map of the peak amplitudes extracted from the FFT:* **a** *amplitude of the first harmonic f_{SAW},* **b** *amplitude of the second harmonic $2f_{SAW}$ for P_{rf} = 31 dBm.* **c** *Weight of f_{SAW} and $2f_{SAW}$ contributions - False color maps of M for P_{rf} = 31 dBm.*

7. Modulation ratio as a function of P_{rf}:

The ratio between the two harmonics amplitudes, $M = \frac{A_2 - A_1}{A_1 + A_2}$, is plotted in false color maps for complete driving electrical power sweep at the IDT in Figure S 9. Like stated in the main text, the second harmonic modulation becomes stronger with increasing P_{rf} and tends to be located close to defects on the SAW path and enables the precise detection of these.

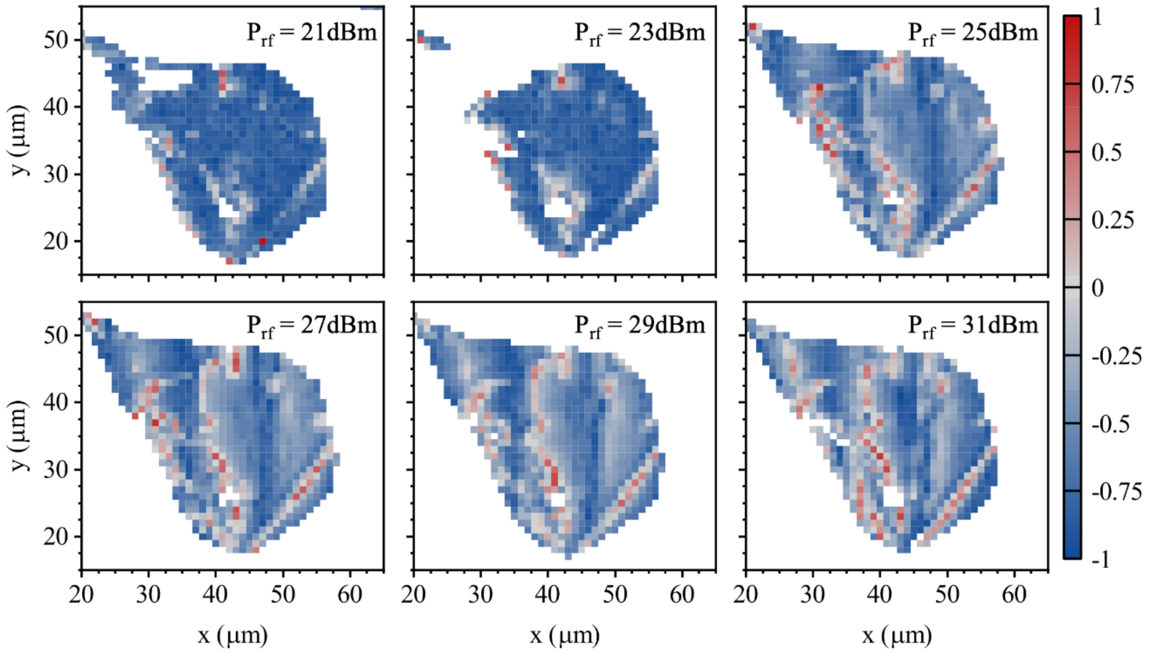

Figure S 9 – P_{rf}-dependence of M – False color maps of the ratio between the two frequency contributions M as a function of the applied radiofrequency power P_{rf} from 21 dBm to 31dBm in 2dB steps.
